# Developmental characteristics of aggregated lymphoid nodules area in the abomasum of fetal Bactrian camels* (Camelus bactrianus)*

**DOI:** 10.1186/s12917-024-04000-3

**Published:** 2024-04-25

**Authors:** Jia Lu, Yu-jiao Cheng, Xiao-hong Xu, Lin-jiang Zhang, Zhi-hua Chen, Lei Liu, Wen-hui Wang

**Affiliations:** 1 College of Veterinary Medicine, Gansu Agricultural University, Lanzhou, 730070 Gansu China; 2 Gansu Provincial Center for Disease Control and Prevention, Lanzhou, 730070 Gansu China; 3 Tianjin Customs District P.R. China, Tianjin, 300041 China

**Keywords:** Abomasum, Aggregated lymphoid nodules area (ALNA), Fetal Bactrian camels, Developmental characteristics

## Abstract

**Background:**

Bactrian camel is one of the important economic animals in northwest China. They live in arid desert, and their gestation period is about 13 months, which is longer than other ruminants (such as cattle and sheep). The harsh living conditions have made its unique histological characteristics a research focus. Aggregated lymphoid nodules area (ALNA) in the abomasum of Bactrian camels, as one of the most important sites for the induction of the immune response, provide a comprehensive and effective protective role for the organism, and their lack of information will affect the feeding management, reproduction and epidemic prevention of Bactrian camels. In this study, the histological characteristics of the fetal ALNA in the abomasum of Bactrian camels at different developmental gestation have been described by using light microscopy and histology .

**Results:**

The ALNA in the abomasum of the Chinese Alashan Bactrian camel is a special immune structure that was first discovered and reported by Wen-hui Wang. To further establish the developmental characteristics of this special structure in the embryonic stage, the abomasum ALNA of 8 fetuses of Alashan Bactrian camels with different gestational ages (5~13 months) were observed and studied by anatomy and histology. The results showed that the aggregation of reticular epithelial cells (RECs) surrounded by a very small number of lymphoid cells was detected for the first time in the abomasum of fetal camel at 5 months gestation, which was presumed to be primitive ALNA. At 7 months gestation, the reticular mucosal folds region (RMFR) appeared, but the longitudinal mucosal folds region (LMFR) was not significant, and histological observations showed that there were diffusely distributed lymphocytes around the RECs. At 10months gestation, RMFR and LMFR were clearly visible, lymphoid follicles appeared in histological observation, lymphocytes proliferated vigorously. By 13 months, the volume of lymphoid follicles increased, forming the subepithelial dome (SED), and there was a primitive interfollicular area between the lymphoid follicles, which contained high endothelial vein (HEV), but no germinal center (GC) was found. In summary, ALNA of Bactrian camels is not fully mature before birth.

**Conclusions:**

Generally, the small intestine PPs of ruminants (such as cattle and sheep) is already mature before birth, while the ALNA in the abomasum of Bactrian camels is not yet mature in the fetal period. During the development of ALNA in Bactrian camel, the development of lymphoid follicles extends from submucosa to Lamina propria. Interestingly, the deformation of FAE changes with age from simple columnar epithelium at the beginning of pregnancy to Simple cuboidal epithelium, which is opposite to the FAE deformation characteristics of PPs in the small intestine of fetal cattle and sheep. These results are the basis of further research on the specificity of ALNA in the abomasum of Bactrian camels.

## Background

Aggregated lymphoid nodules are mainly distributed in the ileum, cecum and tonsils of animals and humans [1–9]. It is a lymphoid tissue formed by the aggregation of lymphoid follicles, which contains a large number of lymphocytes and other Immunocompetent cells [10–13]. It is an important part of mucosal associated lymphoid tissue (MALT). It participates in the first line of defense against pathogen invasion and is an important antigen induction site [14–17] .

According to the 2020 statistics of the Food and Agriculture Organization of the United Nations (FAO), there are 405,000 bactrian camels in China, mainly distributed in Gansu, Qinghai, Xinjiang and Inner Mongolia. Because of its use of labor, meat, hair and milk, and its good adaptability to extremely desolate, barren and harsh environment, the Bactrian camel has incomparable advantages over other animals. It is called "multipurpose livestock" and is a vulnerable animal of the International Union for Conservation of Nature (IUCN) [18]. Over a long period of development and evolution, it has gradually acquired anatomical and biological characteristics that are unmatched by other animal species. Its digestive tract, particularly its stomach, which differs from that of other ruminants demonstrates its morphological adaptability [19–22]. Wen-hui Wang's research on the histology and histochemistry of different parts of the stomach in Bactrian camels suggests that the stomach of Bactrian camels belongs to a special type of ruminant stomach [23], consisting of three chambers: the first and second chambers are the anterior stomach, and the third chamber is the abomasum. This nomenclature has now been more generally accepted [19]. Previous studies have focused on the anatomy and histology of the camel stomach as a digestive and water-storage organ. However, Wen-hui Wang found that the camel stomach not only has a special digestive structure, but also has special immunomorphological features [23]. Under normal conditions, there is very little lymphoid tissue in the animal's stomach and only a small amount of diffuse lymphoid tissues and lymphoid follicles are diffusely distributed. Therefore, there is generally no visible mucosal-associated lymphoid tissue present in the stomach. However, in her study of the immune system of the digestive tract of the Bactrian camel, Wen-hui Wang found that a triangular band of aggregated lymphoid nodules was present in the abomasum, known as ALNA [23]. Like the aggregated lymphoid nodules of other animals' ileum, ALNA is clustered and arranged in the submucosa, forming mucosal folds together with the mucosa. The area of these mucosal folds are large, and the boundary between it and the non-ALNA is very clear. In addition, a large amount of diffuse lymphoid tissue is present in the area between the mucosal epithelium and the glands of the lamina propria, with some isolated lymphoid nodules scattered in the lamina propria and submucosa. Thus, the ALNA greatly increases the storage of lymphoid tissue in the camel's stomach, and its discovery presents new immunomorphological support for further studies of the Bactrian camel's strong resistance to disease.

As a unique immune structure of gastric mucosal organogenesis, it has not been reported in other animals and humans so far. The structure of ALNA is a long triangle belt, located behind the gastric neck and distributed along the lesser curvature of the abomasum. Its mucosal fold is wider and thicker than its surrounding area, forming an obvious boundary with the surrounding non aggregated lymphoid nodules area. According to the different forms of mucosal fold, it can be divided into RMFR and LMFR [23]. Its anatomical characteristics are very different from the Peyer's patches (PPs) in the small and large intestine of Bactrian camels, and also from the PPs in the intestine of other animals [24–29]. In the previous research work of our team, there were research results indicating that ALNA gradually increases before the puberty of Bactrian camel, reaches the most developed period in its life, and then gradually decreases and shrinks, but it still exists at the age of 20 and does not disappear completely [29, 30].The regularities of postnatal development of ALNA is similar to the PPs of Bactrian camel intestine, and the development pattern is also similar to the bursa of Fabricius [30]. In addition, except that the development degree of germinal center of lymphoid nodule is not related to age, the morphology, distribution and number of lymphoid nodule in the folds of abomasal mucosa, as well as the distribution characteristics of reticular fibers and important effector cells, change regularly with age [10, 31].

The above research found that there is a close correlation between the anatomical and histological characteristics of ALNA and age, and its gradual atrophy with age belongs to a physiological atrophy phenomenon [29, 30]. Moreover, those studies have focused on the structure and developmental characteristics of ALNA in Bactrian camels after birth, but the morphological changes during embryonic development have not been studied. Therefore, this study aims to explore the developmental timing of the ALNA in Bactrian camels and its changes in anatomical and histological structures at different stages of embryonic development. The following indicators were specifically studied and observed: the timing and location of the appearance of RECs, the location and growth direction of lymphoid follicles, the nodes of occurrence of the Follicular associated epithelium (FAE) and the interfollicular area, and the cell type changes that occur in the FAE with increasing gestational age. Through the study of these contents, the aim is to provide information for further revealing the mechanism of physiological atrophy in Bactrian camels and clarifying their functions. Meanwhile, as current research on the development of gastrointestinal related lymphoid tissue mainly focuses on the development of various animals, this study will also enrich and expand the data on the development of the digestive mucosal immune system.

## Results

### 5 months gestational age

**Fig. 1:**
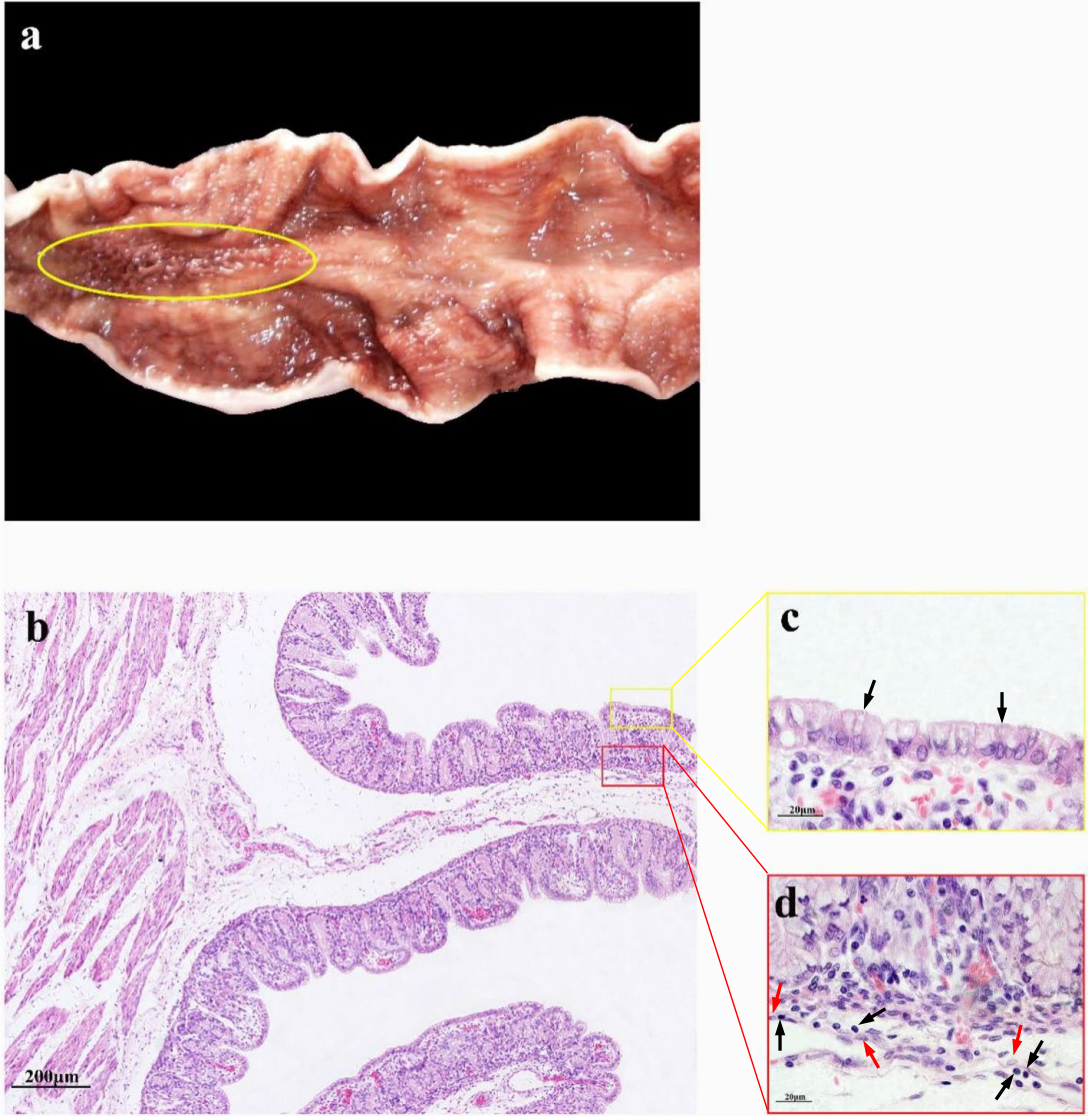
ALNA of Bactrian camel fetus at 5 months of gestation. **a** The mucosal folds of ALNA (yellow oval area) are low and very thin. **b** Under low magnification, there are low mucosal folds. In the submucosa, no lymphocytes aggregation was found. H&E stain, Scale bar 200 μm. **c** The lamina propria of ALNA is composed of diffuse connective tissue, and the mucosal epithelial cells are monolayer columnar epithelial cells (black arrows). H&E stain, Scale bar 20 μm. **d** A small number of lymphoid cells (black arrows) and lightly stained RECs (red arrows) were found in the submucosa and muscularis mucosa. H&E stain, Scale bar 20 μm

In the ALNA of the fetuses at 5 months of gestation, folds can be seen in the area of gastric mucosa marked by yellow oval (Fig. 1a), but the folds are low and very thin (Fig. 1b), and there is little difference in height and thickness between the folds and the surrounding non-ALNA. At high magnification, the mucosal epithelium was columnar epithelium without intraepithelial lymphocytes (Fig. 1c); It was found that the lightly stained RECs in the submucosa and muscularis of mucosa were aggregated, with a few lymphocytes, which was speculated to be developing ALNA (Fig. 1d).

### 7 months gestational age

**Fig. 2:**
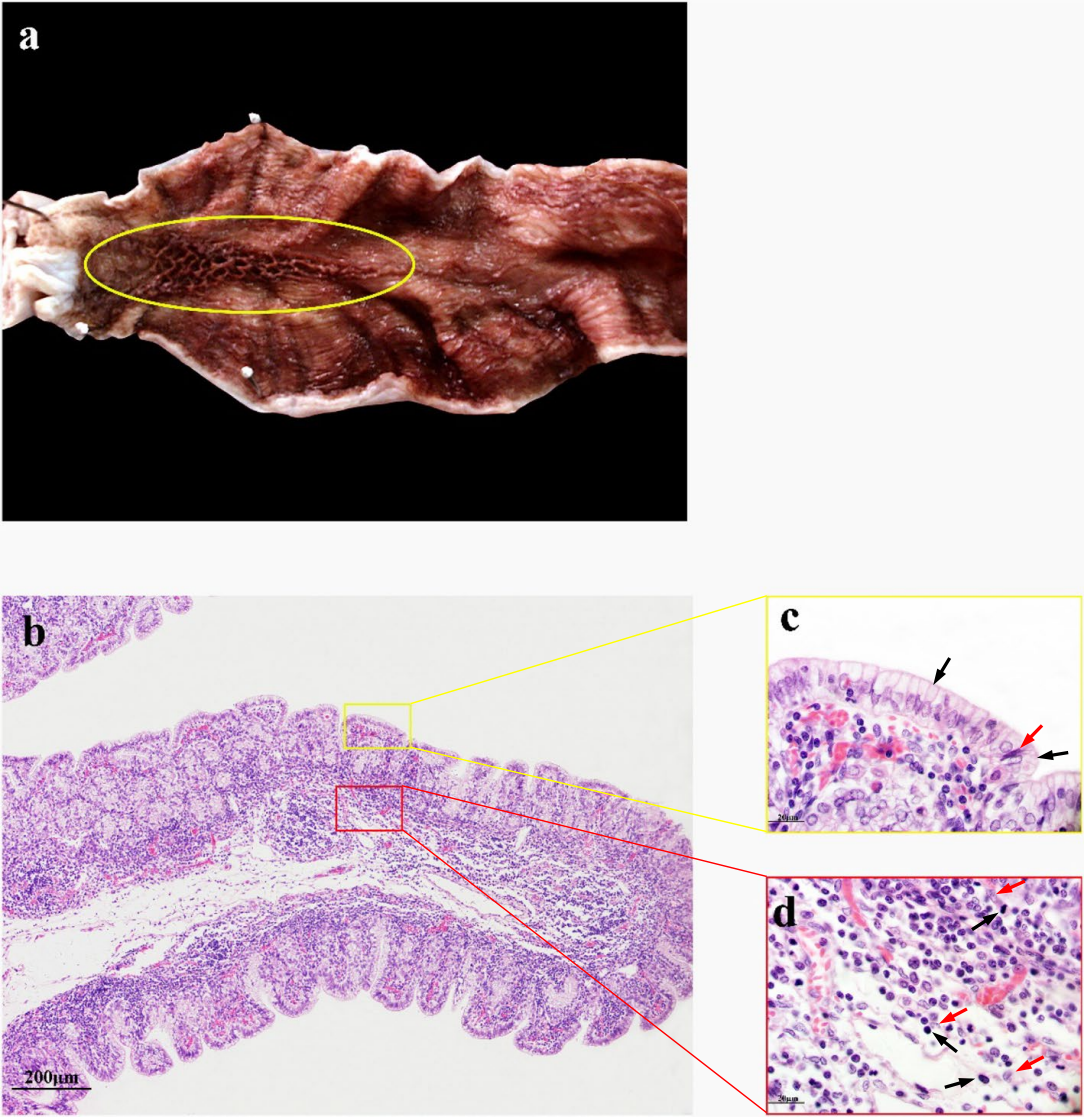
ALNA of Bactrian camel fetus at 7 months of gestation. **a** The ALNA (yellow oval area) mucosal structure of the fetus at 7 months of gestation is relatively clear, and the mucosal fold is higher and thicker than the surrounding non-ALNA. It is mainly composed of RMFR, and the LMFR fold structure is not significant. **b** At low magnification, lymphocytes can be seen in the Submucosa and muscularis mucosa, which are more and more dense than those at 5 months of gestation. H&E stain, Scale bar 200 μm. **c** The mucosal epithelial cells here are monolayer columnar epithelial cells (black arrows), with a small number of intraepithelial lymphocytes (red arrows). H&E stain, Scale bar 20 μm. **d** In the submucosa and muscularis mucosa, some deeply stained lymphocytes (black arrows) were dispersed around the reticular epithelial cells (red arrows), but there was still no formation of lymphoid follicles. H&E stain, Scale bar 20 μm

In the ALNA of the fetuses at 7 months of gestation, the mucosal folds here are higher and thicker than the surrounding areas.Compared with fetuses at 5 months of gestation, the mucosal structure is relatively clear and mainly composed of RMFR, and the LMFR is not significant (Fig. 2a). Histological studies have shown that under the same microscope magnification (100×), The mucosal folds of ALNA at this time were thicker than those at 5 months of gestation, and the lymphocyte density was significantly increased, which was diffused in the submucosa and muscularis mucosae (Fig. 2b). Mucosal epithelial cells are still columnar epithelial cells with some intraepithelial lymphocytes (Fig. 2c); Low density lymphoid cells appear around RECs, mainly distributed in the submucosa and muscularis mucosa (Fig. 2d).

### 10 months gestational age

**Fig. 3:**
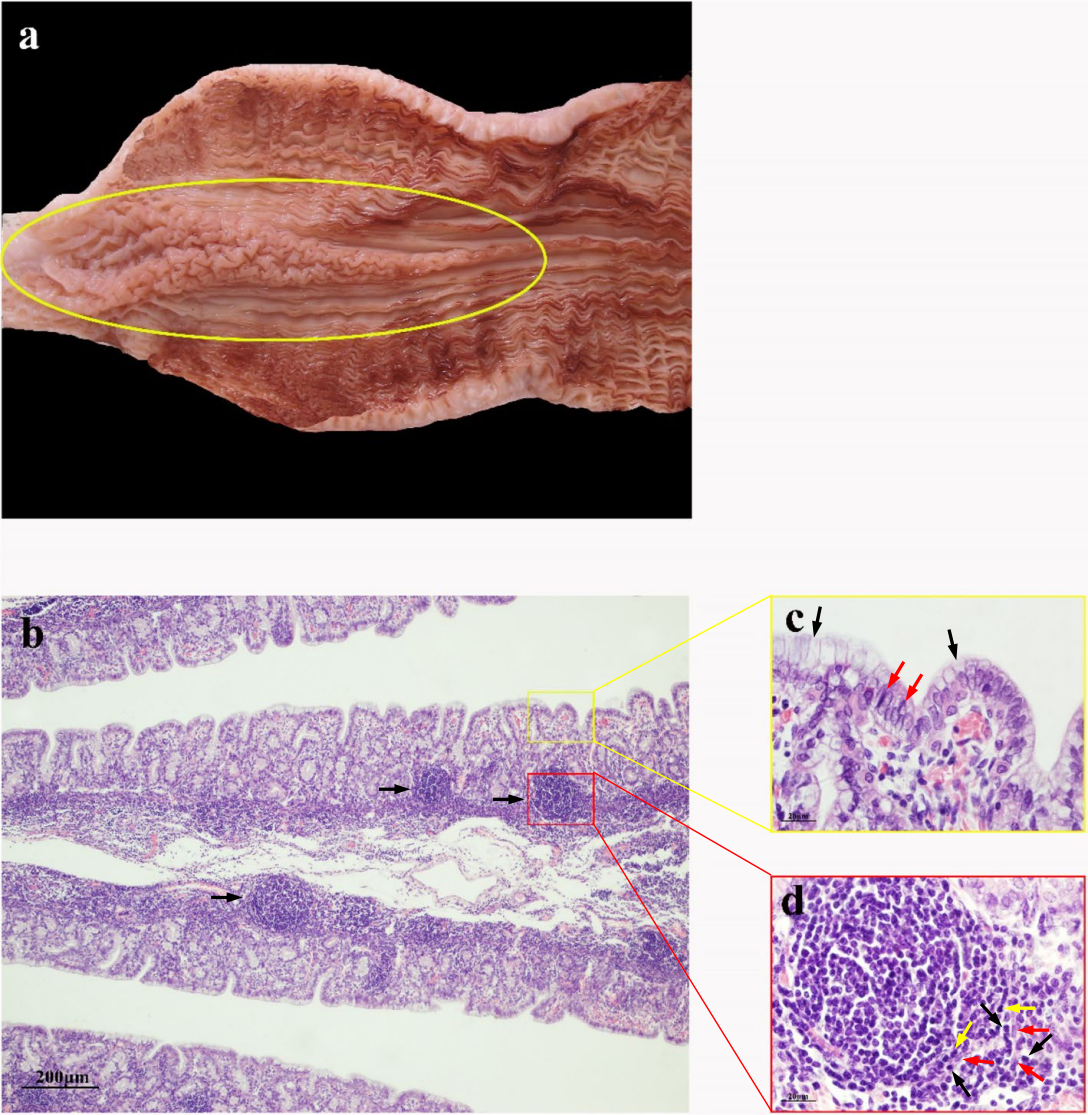
ALNA of Bactrian camel fetus at 10 months of gestation. **a** the mucosal structure of ALNA (yellow oval area) in the abomasum of Bactrian camel fetus at 10 months of gestation is clearer, and the RMFR and the LMFR are also clearly visible, forming a clear boundary with the surrounding non-ALNA. **b** Diffusely distributed lymphoid follicles (black arrows) penetrate the muscularis mucosa and extend to the lamina propria. HE, *Bar* 20 μm. **c** FAE is columnar epithelium (black arrows) with intercellular lymphocytes (red arrow). H&E stain, Scale bar 20 μm. **d** Some deeply stained lymphocytes and lightly stained RECs clustered into lymphoid follicles, which contained mitotic cells (red arrows). H&E stain, Scale bar 20 μm

At 10 months of gestation, the mucosal structure of ALNA was clearer. Next to the RMFR with grid shaped mucosal folds, the LMFR with long protruding mucosal folds begin to appear, but their boundaries are not clear enough to distinguish (Fig. 3a). At this stage, lymphocytes infiltration is dense, and these lymphocytes form lymphoid follicles, which extend through the muscularis mucosa to the lamina propria, and are diffusely distributed in all mucosal folds in the RMFR and the LMFR (Fig. 3b), this is the most significant feature in fetal ALNA at this time. FAE is columnar epithelium (Fig. 3c). Among the follicular cells, there are RECs, and the rest are small to large lymphocytes, some of which are proliferating (Fig. 3d).

### 13 months gestational age

**Fig. 4:**
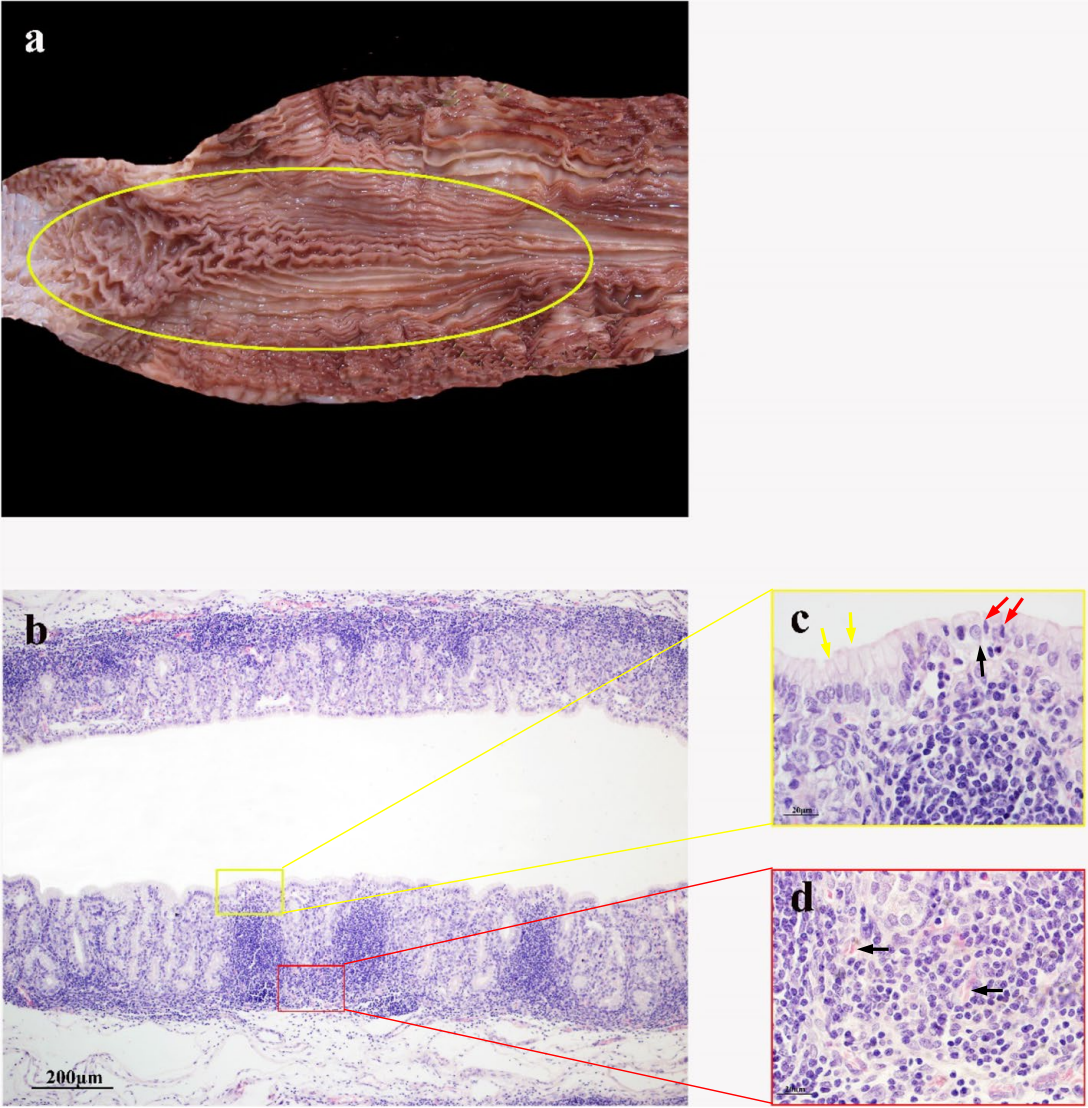
ALNA of Bactrian camel fetus at 13 months of gestation. **b** The mucosal folds of ALNA (yellow oval area) increased and thickened, and developed rapidly. **b** The lymphoid follicles were enlarged in volume and covered by FAE, forming a typical SED structure. H&E stain, Scale bar 200 μm. **c** FAE cells are mainly cuboidal epithelial cells (black arrows), with some intraepithelial lymphocytes (red arrows) scattered between them, while non-FAE cells are mainly monolayer columnar epithelium (yellow arrows). H&E stain, Scale bar 20 μm. **d** The primitive interfollicular area is formed between lymphoid follicles, which contains HEVs (black arrows). H&E stain, Scale bar 20 μm

At 13 months of gestation, the mucosal fold features of RMFR and LMFR are clearly visible, and the boundaries between the two regions are clearly distinguishable. (Fig. 4a). lymphocyte infiltration was more intensive, and these cells had formed follicles with complete structure (Fig. 4b). At this stage, a typical primitive SED was formed. FAE cells are mainly cuboidal epithelial cells with round nuclei in the middle of the cells. The mucosal epithelium other than the FAE is mainly composed of single-layer columnar epithelial cells. Therefore, the follicular dome epithelium is lower than its surrounding area. In addition, there are individual intraepithelial lymphocytes scattered between these cuboidal epithelial cells (Fig. 4c). The primitive interfollicular area begins to form between the lymphoid follicles. There are many HEVs in the primitive interfollicular area, and some lymphocytes are distributed in the tube wall and lumen (Fig. 4d).

## Discussion

It is reported that ileal PP of mouse and human, jejunal PP and ileal PP of bovine, sheep and pigs begin to develop before birth, but some mature before birth (such as jejunal PP and ileal PP of cattle and sheep), while others can fully mature after birth (such as jejunal PP and ileal PP of pigs and mice, as well as human ileal PP) [1, 32–37].In addition, PP of some animals began to develop after birth, such as cecal PP and jejunal PP of chickens [38]. At present, the relevant data on the development of PP in Bactrian camel are not perfect. Therefore, this study mainly refers to the data of bovine, sheep and other animals that have been studied. Reynolds reported that the first primary lymphoid follicle was found in the jejunum of sheep fetuses at about 60 days of gestation. The first ileal PP was formed in the middle of gestation (the 75th day of gestation), but PP finally matured in the last month of gestation. In the last month of gestation, the secondary lymphoid follicles of sheep fetus are composed of three areas, namely SED, GC and interfollicular area. Also, in the last month of gestation, the formation of these three areas marks the maturity of PP [39]. Beyaz et al. reported after the study of bovine fetal ileal PP that the first primordial PP was formed on the 164th day of gestation, began to form the aggregation of lymphoid follicle on the 227th day, and completed the development of follicles on the 271th day to form mature follicles [40]. Based on the results of the above studies, we can know that PP in the small intestine of cattle and sheep begins to develop in the early stages of gestation and matures in the later stages. In this study, we researched the development of ALNA in the fetuses of Alashan Bactrian Camel, and described the development characteristics of ALNA in fetuses of different gestational ages in detail. Pale-staining RECs were clustered in the submucosa and muscularis mucosa of ALNA in the 5-month-old Bactrian camel fetus, and there were a few lymphocytes nearby. At this stage, the ALNA began to develop. Diffuse lymphocytes began to form in the ALNA’s muscularis mucosa of the 7-month-old fetus, and the ALNA was further developing. In the ALNA of a 10-month-old fetus, lymphocytes from the muscularis mucosa converge to form primordial lymphoid follicles. As the number of aggregated lymphocytes gradually increases, primordial lymphoid follicles are formed that gradually increase in volume and extend from the muscularis mucosa into the lamina propria. At 13 months of gestation, the lymphoid follicles in the fold of ALNA’s mucosa began to gather with each other, forming the interfollicular area and FAE, but there was no typical GC. According to the experimental results of this study, the ALNA of Bactrian camels began to develop at the embryonic stage, but did not mature before the birth. Therefore, the results of this study confirmed that the developmental characteristics of the ALNA in Bactrian camel were not exactly the same as those of ileum PP in bovine, sheep and goats [41, 42]. In other words, although the ALNA of Bactrian camels begins to develop at the embryonic stage, like the small intestinal PP of cattle, goats and sheep, it does not develop as fast as the small intestinal PP of these animals. Until birth, the ALNA of Bactrian camels is still in an immature state. We speculate that the mature node of ALNA in Bactrian camel is likely to be in the postnatal period, and its maturation may require stimulation of breast milk or antigen.

It has been shown that the lymphoid follicles of PP in animals and humans form in the submucosa, then pass through the muscularis mucosa and extend to the lamina propria mucosa; However, some researchers believe that the lymphoid follicles of PP initially form in the lamina propria of the mucosa, and then extend into the submucosa through the muscularis mucosa. The above views are based on the results of the study of PP structure in adult animals. Asari et al.(1987) reported that the primary lymphoid follicles of the ileum of bovine fetus first appeared in the submucosa in the form of aggregation formed by small lymphocytes and a small number of cytosolic basophilic cells, and then some lymphoid follicles gradually developed through the mucosal muscularis to reach the mucosal surface to form the SED and FAE [37]. Ishino et al.(1991) reported that the first primary lymphoid follicles of cattle appeared in the lamina propria [43]. Reynolds et al. (1983) reported that the first primary lymphoid follicle of sheep appeared in the lamina propria of the mucosa, and with the development of the sheep fetus, the number and volume of lymphoid follicles increased gradually. In the last month of gestation, the volume of those lymphoid follicles had expanded, crossing through the muscularis mucosa and producing a thickening in the submucosa [39]. Beyaz et al. (2004) found that, consistent with the results of sheep and goat fetuses, the first primordial dome structure of bovine fetuses was formed in the lamina propria, but later, lymphoid follicles penetrated through the muscularis mucosa and extended into the submucosa, gradually forming mature PP in the submucosa [40]. In this study, ALNA of Bactrian camels began to develop in the submucosa during the early trimester of pregnancy, which was evidenced by the presence of a few lymphoid cells near the RECs in the submucosa, and these lymphoid cells aggregated around the RECs. In the second trimester of pregnancy, the number of lymphoid cells increased and distributed diffusely around the RECs in the submucosa and muscularis mucosa. In the third trimester of pregnancy, the number of lymphoid cells in submucosa and muscularis mucosae increases continuously, lymphoid cells gather together to form primitive lymphoid follicles, and the volume of primordial lymphoid follicles gradually increases, taking submucosa and muscularis mucosae as the base point, growing and extending to lamina propria. Therefore, the results of this study further support the view that lymphoid follicles extend from the submucosa to the lamina propria in PP.

Follicle associated epithelium (FAE), or dome epithelium, is the epithelial cell covering the top of PP. FAE in adult animals is mainly composed of intestinal epithelial cells and Microfold cells, a special cell distributed between them. Microfold (M) cells have the ability to absorb and transport antigen. Beyaz et al. found that the first primordial FAE of PP in the ileum of bovine fetus was only composed of cuboidal epithelial cells and some cells with a large number of multivesicular bodies in their cytoplasm, which were very similar to M cells in the epithelium related to PP follicles in the jejunum [40]. However, these cuboidal epithelial cells became columnar epithelium during late gestation. In this study, unlike the FAE development characteristics of PP in the ileum of cattle, the FAE of the primordial lymphoid follicles in ALNA of Bactrian camel is mainly composed of columnar epithelial cells. By the 13th month of gestation, with the formation of the SED, the FAE gradually changed into cuboidal epithelium, which was also mixed with some intraepithelial lymphocytes. The result suggests that the developmental characteristics of FAE in ALNA of Bactrian camel are special before birth.

## Conclusions

In this study, based on light microscopy and paraffin-section technique, we first explored and described the developmental characteristics of ALNA—which is a specific organoid lymphoid tissue in Alashan Bactrian camel—during the fetal period.

To sum up, the ALNA in the abomasum of Bactrian camel is not mature in the fetal period, and the primordial lymphoid follicles begin to develop in the late gestation. During the development of ALNA in Bactrian camel, the development of lymphoid follicles extends from submucosa to lamina propria. With the growth of gestational age, ALNA mucosal folds became more and more developed, and the volume and number of lymphoid follicles gradually increased. The histological results were consistent with the anatomical results. These results laid a foundation for further study of the specificity of ALNA mucosal immunity, and provided certain immunomorphological support for the study of the correlation between the development of ALNA in Bactrian camel fetus and mucosal immune function and animal gestational age.

## Materials and methods

All experimental procedures were approved by the Animal Care and Use Committee (IACUC) of the College of Veterinary Medicine of Gansu Agricultural University (Approval No: GSAU-Eth-VMC-2021-032).

### Experimental animal

Eight pubertal healthy pregnant female camels aged 3 to 5 years and with a gestation period of 5 to 13 months (contains 4 different gestational periods: 5 months, 7 months, 10 months, and 13 months. The number of female camels per gestation period is 2), were purchased from a slaughterhouse in Minqin County, Gansu Province. And the gestation month is determined by rectal examination [44]. The female camel was anesthetized intravenously with pentobarbital sodium (20 mg/kg). Wait until the animal reaches a moderate anesthesia state and exsanguinated to death. Cut open the uterus to expose the fetuses and separate them from the female camels. All efforts were made to minimize suffering.

### Tissue sampling

Then use a scalpel along the midline of the abdomen, from the cartilago xiphoidea to the navel, to cut the abdomen of the fetus of the Bactrian camel, expose the abomasum, cut off the two ends of the abomasum— the cardia and pylorus, and remove the entire abomasum. The abomasum was cut along one side of the major curvature.

### Anatomical observation

The gastric contents were removed, and the mucosal surface was thoroughly rinsed with physiological saline. The following indicators were carefully observed: Anatomical location of ALNA, Characteristics of ALNA mucosal surface at different gestational ages, anatomical characteristics and differences between the mucosal folds of RMFR and LMFR, differences in the characteristics of ALNA and surrounding non-ALNA mucosal surfaces.

After the observation, the abomasum, which had been removed and cut along the greater curvature of the abomasum, was laid flat with the mucosa side up and the plasma side down on a thick dark tarpaulin and secured with a large headpin along the cut edge. Using a Nikon Coolpix 4500 digital camera with the lens pointing downwards vertically, anatomical photographs of the ALNA in Bactrian camels of different gestational ages were taken.

### Histological studies

Samples were quickly obtained from the densest area of ALNA folds in the recently deceased fetuses of Bactrian camels. The histological materials were fixed in 10% neutral formalin solution, and then paraffin sections were made and stained with hematoxylin and eosin. The shape, distribution and number of lymphocytes and lymphoid follicles, as well as the occurrence time and distribution of SED, interfollicular area and HEV were observed under the Orthomosaic Microscope and Imaging System(Brand and model: OLYMPUS BX51; Settings: eyepiece 10×, objective 10× and 100× respectively). 10 sections were observed in each sample.

## Data Availability

The data set supporting the results of this article are available from the author.

## Abbreviations

ALNA: Aggregated lymphoid nodules area
RECs: Reticular epithelial cells
RMFR: Reticular mucosal folds region
LMFR: Longitudinal mucosal folds region
SED: Subepithelial dome
HEV: High endothelial vein
GC: Germinal center
MALT: Mucosal associated lymphoid tissue
IUCN: International Union for Conservation of Nature
PPs: Peyer's patches
FAE: Follicular associated epithelium
